# Artificial barriers prevent genetic recovery of small isolated populations of a low-mobility freshwater fish

**DOI:** 10.1038/s41437-017-0008-3

**Published:** 2018-01-12

**Authors:** R. A. Coleman, B. Gauffre, A. Pavlova, L. B. Beheregaray, J. Kearns, J. Lyon, M. Sasaki, R. Leblois, C. Sgro, P. Sunnucks

**Affiliations:** 1 Applied Research, Melbourne Water Corporation, Docklands, VIC 3008 Australia; 2 School of Biological Sciences, Monash University, Clayton, VIC 3800 Australia; 3 Centre d’Etudes Biologiques de Chizé, UMR 7372, CNRS and Université de La Rochelle, F-79360 Beauvoir sur Niort, France; 4 INRA, USC 1339 CEBC, F-79360 Villiers en Bois, France; 5 Molecular Ecology Lab, Flinders University, Adelaide, SA 5001 Australia; 6 Arthur Rylah Institute for Environmental Research, Department of Environment, Land, Water and Planning, Heidelberg, VIC 3084 Australia; 7 CBGP, INRA, CIRAD, IRD, Montpellier SupAgro, University of Montpellier, Montpellier, France; 8 Institut de Biologie Computationnelle, University of Montpellier, Montpelier, France

## Abstract

Habitat loss and fragmentation often result in small, isolated populations vulnerable to environmental disturbance and loss of genetic diversity. Low genetic diversity can increase extinction risk of small populations by elevating inbreeding and inbreeding depression, and reducing adaptive potential. Due to their linear nature and extensive use by humans, freshwater ecosystems are especially vulnerable to habitat loss and fragmentation. Although the effects of fragmentation on genetic structure have been extensively studied in migratory fishes, they are less understood in low-mobility species. We estimated impacts of instream barriers on genetic structure and diversity of the low-mobility river blackfish (*Gadopsis marmoratus*) within five streams separated by weirs or dams constructed 45–120 years ago. We found evidence of small-scale (<13 km) genetic structure within reaches unimpeded by barriers, as expected for a fish with low mobility. Genetic diversity was lower above barriers in small streams only, regardless of barrier age. In particular, one isolated population showed evidence of a recent bottleneck and inbreeding. Differentiation above and below the barrier (*F*_ST_ = 0.13) was greatest in this stream, but in other streams did not differ from background levels. Spatially explicit simulations suggest that short-term barrier effects would not be detected with our data set unless effective population sizes were very small (<100). Our study highlights that, in structured populations, the ability to detect short-term genetic effects from barriers is reduced and requires more genetic markers compared to panmictic populations. We also demonstrate the importance of accounting for natural population genetic structure in fragmentation studies.

## Introduction

Widespread loss of habitat and associated fragmentation of wildlife populations is a major threat to global biodiversity (Sala et al., 2000; Foley et al., 2005; Fischer and Lindenmayer, 2007). Fragmentation often results in small, isolated populations that are more vulnerable to stochastic events. For example, fluctuations in climate such as drought, natural catastrophes such as wildfires and demographic variation such as annual breeding success. As a consequence small, isolated populations are vulnerable to loss of genetic diversity through genetic drift (Fischer and Lindenmayer, 2007). Loss of genetic diversity contributes to increased extinction risk for small populations, because it reduces the potential of populations to adapt to future environmental changes such as disease, pollutants, and climate change, and can result in loss of fitness through inbreeding and fixation of deleterious alleles (Frankham, 2005; Willi et al., 2006). Loss of genetic diversity in small, isolated populations has been observed across a broad range of taxonomic groups including mammals, birds, reptiles, and fishes (Frankham, 1996; Taylor et al., 2003; Whiteley et al., 2013; Rivera-Ortíz et al., 2014). Where population isolation and loss of genetic diversity through drift threatens the viability of small populations, the managed movement of individuals into these populations from a suitable source population (assisted gene flow) can rapidly increase genetic diversity and improve population fitness (Frankham, 2015; Whiteley et al., 2015). Thus, assessing levels of genetic diversity and the strength of genetic drift after population isolation can assist in developing effective conservation strategies.

Freshwater ecosystems are especially vulnerable to habitat loss and fragmentation, given the linear nature of rivers and streams and the need for many aquatic organisms to undertake longitudinal (upstream and downstream) and lateral (notably onto adjacent floodplains during high flows) movements as a part of their life history. Along with other threats, such as overexploitation, water pollution, flow modification, and invasion by exotic species, these aspects of freshwater ecosystems have contributed to a proportionally higher number of threatened freshwater species per area compared to terrestrial and marine ecosystems (Dudgeon et al., 2006; Strayer and Dudgeon, 2010). Artificial barriers to the movement of freshwater organisms include dams, pipes, culverts, weirs, levees, altered flow regimes, and aquatic pollution, with many freshwater systems affected worldwide (Jackson et al., 2001; Nilsson et al., 2005). Here we use the term “barrier” to mean any restriction to the movement of individuals and their genes. The negative impacts of barriers on fish distribution and abundance have been well documented, and with >60% of the world’s 227 largest rivers classified as highly fragmented, barriers are recognised as a major threat to freshwater fishes (Lucas et al., 2001; Reid et al., 2013; Hansen et al., 2014). In some cases, fragmentation due to dams has led to extirpation of populations, while in others it has exerted negative genetic effects by restricting gene flow and causing population declines (Angermeier, 1995; Faulks et al., 2011; Hansen et al., 2014).

To understand the threats associated with barriers to freshwater fish communities and find appropriate management solutions, it is important to evaluate the genetic consequences of barriers for species with a range of mobility. While several studies have assessed the impact of natural or artificial barriers on genetic structure and diversity of freshwater fish species that have the potential to move substantial distances throughout their life, especially salmonid species (for example, Morita and Yamamoto, 2002; Costello et al., 2003; Taylor et al., 2003; Wofford et al., 2005; Beneteau et al., 2009; Whiteley et al., 2013; Gouskov et al., 2016), studies of non-salmonid and low-mobility species are comparatively rare in the fragmentation literature (Dehais et al., 2010; Roberts et al., 2013; Lean et al., 2017). Assessing the impact of recent barriers on genetic structure is challenging for species with low mobility: when mating occurs preferentially within areas that are small relative to the global population range, genetic differentiation can increase across the landscape with distance (isolation-by-distance (IBD), Wright, 1943). In such structured populations, the rate of loss of genetic variability globally is reduced compared to a panmictic population of the same size (Leblois et al., 2006), and so it takes more generations for the genetic signature of a barrier effect to develop (Landguth et al., 2010). To avoid erroneously attributing pre-existing genetic structure to barrier effects, background levels of spatial genetic structure must be considered.

In this study, we focus on the impacts of artificial barriers on the genetic structure and diversity of a non-migratory freshwater fish species, the southern river blackfish, *Gadopsis marmoratus*. As well as having low mobility, this highly territorial species has low fecundity, making its small populations highly vulnerable to environmental stochasticity (Jackson et al. 1996; Huey et al., 2017). Over much of its range, the distribution of *G. marmoratus* is contracting and population sizes are declining (Morris et al., 2001; Lintermans, 2007; Hammer et al., 2009; Huey et al., 2017; Unmack et al., 2017). Recent genetic studies of *G. marmoratus* across the Murray-Darling Basin (Lean et al., 2017) and in northern south-eastern Australia (Huey et al., 2017) showed low local genetic diversity, suggestive of limited capacity of small populations to adapt to future environmental changes. In addition, strong genetic structure was found at both large and small spatial scales, suggestive of low gene flow across sites and strong effects of genetic drift. While no IBD was detected in both studies, it was suggested that patterns of IBD could be detected over the spatial scale of a stream or catchment unimpeded by barriers. It remains unclear whether the observed genetic structure in *G. marmoratus* is a natural outcome of low dispersal or a result of the combined effect of low dispersal and habitat fragmentation. Here we test for an effect of artificial barriers on genetic structure in *G. marmoratus* in south-eastern Australia at a small spatial scale (from 1.5 to 15 km within individual streams) after accounting for natural genetic structure. The upper Yarra River system near Melbourne, Victoria, Australia presents an excellent opportunity to design an assessment of the impacts of barriers of known age on the genetic structure of a low-mobility freshwater fish. In response to Melbourne’s human population growth, a network of stream flow diversion weirs of known age has progressively increased in extent in the upper Yarra River system, with staged installation of water supply weirs and dams since 1893.

We aimed to test the hypothesis that isolation of a low-mobility freshwater fish by artificial barriers increases genetic drift upstream, resulting in (1) stronger differentiation between populations above and below barriers than would be expected under natural processes, and (2) reduced genetic diversity and inbreeding within populations above barriers compared to populations below barriers. More specifically, we predicted that loss of genetic diversity in populations above each barrier and genetic differentiation across the barrier would depend on (1) the age of the barrier, (2) the size of the above-barrier population (based on catchment area) and (3) the disturbance history (for example, wildfire and severe drought) that could lead to bottlenecks compounding genetic drift due to fragmentation. To achieve our aims, we analysed microsatellite DNA marker data for populations above and below water supply barriers in five streams, where barrier ages were 45–120 years old (~9–24 *G. marmoratus* generations) and catchment sizes were 14–337 km^2^. To assess our ability to distinguish barrier effects from background genetic structure, we first estimated the background levels of differentiation across sample sites within connected parts of the catchment. We then simulated the effects of genetic drift on genetic diversity and differentiation (*F*_ST_) across a barrier for different densities of populations with background genetic structure similar to those estimated in our observed data set. We also used simulations to investigate whether barrier effects on genetic differentiation were masked by unidirectional downstream gene flow (Dehais et al., 2010; Roberts et al., 2013).

## Materials and methods

### Study area

**Fig. 1:**
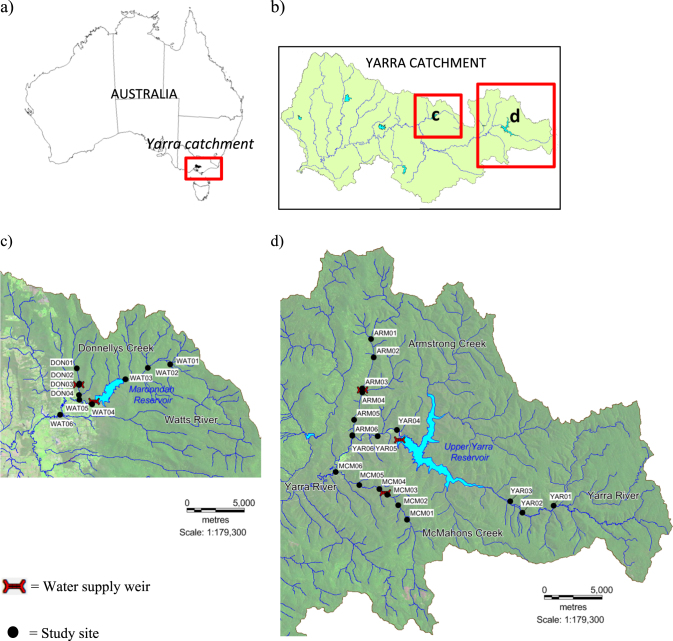
Yarra River catchment, the location of water supply weirs and study sites. ARM Armstrong Creek, DON Donnellys Creek, MCM McMahons Creek, WAT Watts River, YAR Yarra River

The Yarra River catchment, Victoria, Australia, covers a total area of over 4000 km^2^, with our study area comprising two distinct sub-catchments: the Watts River sub-catchment (including Donnellys Creek) and the upper Yarra River sub-catchment (including Armstrong and McMahons creeks) (Fig. 1). Land use in the Watts River sub-catchment is 81.0% forested, 15.1% rural and 3.9 % urban, and in the upper Yarra River sub-catchment is 99.3% forested and 0.7% rural. There are also two distinct stream types: rivers with large catchments and reservoirs (upper Yarra River 337 km^2^, Upper Yarra Reservoir–capacity 200,579 megalitres (ML) and surface area 777 ha; Watts River 165 km^2^, Maroondah Reservoir–22,179 ML, 199 ha) and creeks with small catchment areas and diversion weirs (Donnellys Creek–14 km^2^, McMahons Creek–44 km^2^ and Armstrong Creek–55 km^2^) (Table 1). At the time of this study, the ages of water supply structures in the upper Yarra River system were 45–120 years, with Donnellys Creek weir being the oldest, constructed in 1893. Maroondah Reservoir in the Watts River was constructed in 1927, Upper Yarra Reservoir in the Yarra River in 1959, and Armstrong Creek and McMahons Creek weirs in 1968. The heights of these structures range from ~1.5 m (Donnellys Creek weir) to 91 m (Upper Yarra dam), with heights for Maroondah dam on the Watts River, Armstrong weir, and McMahons weir being ~41 m, 4.5 m and 2.7 m, respectively. Although occasional downstream migrations may occur over the smaller barriers (namely, Donnellys, McMahons, and Armstrong creeks), all barriers were expected to permanently restrict upstream movement for the study species. The main disturbance events that occurred since installation of the water supply barriers were droughts and wildfires. Significant droughts that affected the entire study area were during 1967–1968, 1982–1983, and late 1996–mid 2010. Wildfires in 1983 affected the entire McMahons Creek system, and small areas and Armstrong and Upper Yarra below the barriers (Woodgate, 1984), and wildfires in 2009 resulted in severe burning of substantial areas of the Armstrong Creek catchment above the barrier, and low-severity burning within the Donnellys Creek and Watts River catchments (Feikema et al., 2013).

**Table 1:** Genetic characteristics of *Gadopsis marmoratus* populations above and below barriers in selected waterways within the Yarra River catchment, Victoria, Australia

Waterway	Barrier installed	Barrier height (m)	Upstream or Downstream of Weir	Area above weir (km^2^)	*n*	*N*_a_	*A*_r_	*H*_o_	*H*_e_	HWE *P*-value	*F*_IS_	*N*_e_	*F*_e_
Armstrong	1968	4.5	Upstream	55	40	4.50	4.11	0.476	0.461	0.461	−0.033	98.1 (23.2-Inf)	−0.043
			Downstream		45	5.00	4.53	0.467	0.442	0.032	−0.055	137.7 (49.1-Inf)	
Donnellys	1893	1.5	Upstream	14	30	3.50	3.47	0.516	0.477	0.117	−0.083	10.9 (4.6–25.7)	0.055
			Downstream		30	3.88	3.80	0.542	0.505	0.995	−0.073	10.7 (5.8–19.9)	
McMahons	1968	2.7	Upstream	44	24	2.50	2.50	0.396	0.396	0.078	0.002	8.3 (1.7–205.6)	0.161
			Downstream		44	4.13	3.94	0.472	0.472	0.802	0.000	30.3 (13.5–110.9)	
Watts	1927	41	Upstream	165	45	5.25	4.93	0.507	0.507	0.268	−0.003	Inf (87.4-Inf)	−0.095
			Downstream		29	4.75	4.53	0.397	0.463	0.036	0.144	39.3 (14.1-Inf)	
Yarra	1959	90	Upstream	337	28	4.50	4.33	0.473	0.466	0.861	−0.015	67.5 (15.2-Inf)	−0.112
			Downstream		45	4.88	4.37	0.403	0.419	0.565	0.039	632.5 (52.5-Inf)	

Number of individuals (*n*), average number of alleles per locus (*N*_a_), allelic richness (*A*_r_) corrected for a minimal sample size of 24 individuals, expected (*H*_e_) and observed heterozygosity (*H*_o_), *P*-value of the Hardy–Weinberg Equilibrium (HWE) exact test, *F*_IS_ value, effective population size estimates (*N*_e_, with 95% confidence intervals) based on the LDNe method and effective inbreeding coefficient (*F*_e_).

### Southern river blackfish (Gadopsis marmoratus)

*Gadopsis marmoratus* sensu lato has a range across south-eastern Australia, being widespread in Victoria and present in parts of south east Queensland, eastern New South Wales and south east South Australia (Allen et al., 2002). Differences in morphological features and multiple genetic studies indicate that *G. marmoratus* represents a species complex—most notably between “northern” and “southern” geographic regions (Sanger, 1986; Ovenden et al., 1988; Miller et al., 2004; Ryan et al., 2004; Hammer et al., 2014). The candidate species in this study, the southern-basin lineage (“SBA”), includes Victorian and Tasmanian systems draining to Bass Strait (Hammer et al., 2014; Unmack et al., 2017).

Based on 757 *G. marmoratus* individuals from our study area, Sanger (1986) measured a mean total length of 157 mm (range 27–420 mm) and growth rates of ~40 mm/year for the first 6 years of life. Their lifespan is at least 8 years (Sanger, 1986; Koehn et al., 1994), with sexual maturity of females from 2 years old (Sanger, 1986). On the basis of these sexual maturity and lifespan estimates, we assume a generation time of 5 years for *G. marmoratus*, which corresponds to between 9 and 24 generations of isolation due to the construction of water supply barriers in this study. Annual fecundity is low (usually <500 eggs), increasing with fish size (Jackson, 1978; Sanger, 1986; Jackson et al., 1996). Although movement of all life stages of *G. marmoratus* is poorly understood, adults have a very small home range, usually <30 m of channel length, with occasional longer movements of up to ~200 m (Koehn, 1986; Khan et al., 2004; Koster and Crook, 2008).

### Sample collection

Between 2 April 2012 and 14 March 2013, a total of 366 *G. marmoratus* were caught using a backpack electrofishing unit (Smith-Root, Vancouver, WA, USA) from 28 sites across five streams (Fig. 1; Supplementary Table S1). A total of 24–45 individuals were sampled from 2 to 3 sites above and 2 to 3 sites below each barrier. At each site, backpack electrofishing (Smith-Root Model 20b) was undertaken to sample between 50 and 600 m (mean 200, SE 7) of stream in an attempt to collect up to 15 fish. Although the goal was to have similar distances between sites above and below each barrier, site selection was constrained by suitable access and the length of each stream. River distances between sample sites, calculated with Network Analyst in Arc GIS 10.4 (ESRI), were 1.4–3.8 km (mean 2.2, SE 0.3) above each barrier and 0.9–5.6 km (mean 2.6, SE 0.5) below each barrier. Total reach length surveyed above each barrier (the distance from the uppermost survey site to the barrier) ranged from 1.4 (Donnellys Creek) to 5.4 km (Yarra River), with a mean reach length of 3.9 km (SE 0.8). Total reach length surveyed below each barrier (the distance from the most downstream survey site to the barrier) ranged from 0.9 (Donnellys Creek) to 7.6 km (Watts River), with a mean reach length of 4.6 km (SE 1.1). Instream habitat (for example, stream width, riparian vegetation cover, substratum composition) was primarily homogenous across all sites within each stream, and catchment land cover was dominated by forest, except in the Watts River where the catchment below Maroondah Reservoir has been partially cleared for agriculture and the Healesville township. Given the small spatial scale between sample sites in each stream, historical biogeographic factors are unlikely to be influencing population genetic structure above and below each barrier.

The total length and the total weight of each captured individual were measured and a small piece of caudal fin (~3–5 mm^2^ depending on fish size) was collected and preserved in 100% ethanol prior to being stored in the laboratory at −20 °C. Fish were released after sampling. In this study, total length of *G. marmoratus* was 38–455 mm (mean 216.8, SE 4.7) and weight 1–830 g (mean 136.0, SE 7.2) (see also Supplementary Table S2 in Supplementary Material). To reduce the potential of closely related offspring skewing population assessments, juveniles <60 mm total length (*n* = 5) were removed from genetic analyses.

### Genetic variation

Total DNA was extracted from *G. marmoratus* fin clips using a salting-out DNA extraction protocol (Sunnucks and Hales, 1996) or a DNeasy Blood and Tissue kit (Qiagen, Hilden, Germany). Samples were genotyped using 11 microsatellite DNA markers developed for *G. marmoratus* (Ling et al., 2013) and amplified in two separate multiplex reactions (Plex A and B) following Beheregaray et al. (2004)—see also Supplementary Appendix S1 in Supplementary Material. Genotypes were determined using GeneMapper v4.0 software (Applied Biosystems, Foster City, CA, USA). Three loci previously found to be variable in other *G. marmoratus* populations (*Gama02*, *Gama05*, and *Gama12*) were monomorphic in all samples of this study (Supplementary Table S3 in Supplementary Material) and were removed from subsequent analyses, leaving eight polymorphic loci for statistical analyses. Tests for deviations from Hardy–Weinberg for each locus and linkage equilibria for each locus pair were performed using GENEPOP 4.1 (Rousset, 2008) for each sample site, and for ‘pooled samples’ (the 2–3 sample sites above each barrier in each stream, and the same for below the barrier). This pooling was justified because there was no spatial autocorrelation at this spatial scale (<5.6 km), and even though pooling would tend to increase homozygous excess and linkage disequilibrium, no deviations from Hardy–Weinberg and linkage equilibria were significant after correction for multiple tests (Supplementary Appendices S2 and S3 in Supplementary Material, and Results section). Significance of tests was assessed following a false discovery rate correction for multiple tests (Benjamini and Hochberg, 1995) with a nominal significance level of 5%. Observed and expected heterozygosities (*H*_o_ and *H*_e_) and Weir and Cockerham’s (1984) estimate of *F*_IS_ were calculated using GENEPOP 4.1, for each sample site, and for pooled samples (one pool above each barrier in each stream, and one below). Using the rarefaction procedure implemented in FSTAT 2.9.3.2 (Goudet, 1995, 2001; El Mousadik and Petit, 1996), for each locus we calculated allelic richness corrected for sample size (*A*_r_) for ≥6 individuals at single sample sizes, and ≥24 individuals in the pooled samples.

### Regional genetic structure

To understand the extent to which populations might be interconnected throughout the entire study area, population genetic structure across all sample sites was assessed using Bayesian clustering in STRUCTURE Version 2.2 (Pritchard et al., 2000), with the admixture model and correlated allele frequencies (Falush et al., 2003). To determine the number of clusters (*K*) within the complete data set, ten replicate runs of 2 × 10^6^ Markov chain Monte Carlo (MCMC) iterations, after an initial burn-in period of 5 × 10^5^ iterations, were performed for values of *K* ranging from 1 to 10 (the maximum set to the number of pooled samples). Results were summarised using the standard pipeline on the CLUMPAK web server (Kopelman et al., 2015). The most likely number of clusters (*K*) was explored using the estimated logarithm of likelihood (LnP(D)) and the Evanno et al. (2005) Δ*K* method that finds the point of greatest change in the distribution of LnP(D) with STRUCTURE HARVESTER Version 0.6.92 (Earl and vonHoldt, 2012). We further explored genetic structure within the two main infered clusters by analyzing them independently using the same settings (Supplementary Appendix S2 in Supplementary Material).

### Assessing the background level of genetic structure

To assess the extent of background genetic structure, we performed spatial autocorrelation analyses and IBD tests for below-barrier sample sites that are still connected via the mainstem of the Yarra River (that is, nine below-barrier sites in Armstrong Creek, McMahons Creek and Yarra River, separated by up to 13.2 km). Spatial autocorrelation was investigated with SPAGEDI 1.5 (Hardy and Vekemans, 2002), computing the kinship coefficient of Ritland (1996) to assess genetic similarity among pairs of individuals using 2000-m distance class sizes from 0 to 13.2 km (the maximal distance between two sample sites). The first distance class (0 m) included individuals caught from the same sampling location. For each distance class, significant deviation of spatial autocorrelation patterns from a random distribution of genotypes was tested by 10,000 random permutations of individuals (for the same sampling location) and individual locations (for the other distance classes). IBD was analysed by regressing pairwise estimates of *F*_ST_ /(1−*F*_ST_) against river distance between sample sites (Rousset, 1997), and tested using a Mantel test (10,000 permutations) with GENEPOP 4.1. Similar analyses were not performed for the Watts River sub-catchment due to low number of samples and low sample sizes below barriers.

### Comparing differentiation across barriers to background level of differentiation

To compare genetic differentiation across barriers to background levels of genetic differentiation, we used the pooled samples above and below each barrier within each stream. Using FSTAT, we calculated Weir and Cockerham (1984) pairwise *F*_ST_ values: (1) above versus below each barrier, and (2) between the below-barrier samples in each sub-catchment (Watts River or Upper Yarra). The significance of *F*_ST_ values was determined using 45,000 permutations. We also explored *D*_est_ values, which more accurately account for differences in allelic diversity than does *F*_ST_ (Jost, 2008), calculated with GENALEX Version 6.5 (Peakall and Smouse, 2012). However, since *D*_est_ values did not show different patterns than *F*_ST_, we only report *D*_est_ values but do not discuss them further.

### Assessing the effect of barriers on genetic diversity and inbreeding

To test for differences in genetic diversity above and below each barrier, *A*_r_ was used instead of *H*_e_ because it is more sensitive to recent reductions in population size (Schwartz et al., 2007). Linear mixed models were run using the “*lmer*” function implemented in the *lme4* package (Bates et al., 2015) in R 3.1.3, with *A*_r_ at individual sample sites as the response variable. The location of sample sites relative to barriers (*weir_side*, two levels- above- and below barrier) was included as a fixed factor. Because progressive loss of genetic diversity through drift in small, isolated populations above barriers was expected to depend on: i) population size above the barrier and ii) the number of generations since isolation, we included a fixed factor (catchment_type, two levels) separating the two rivers with large catchments (Yarra and Watts rivers) from the three creeks with small catchments (Donnellys, Armstrong and McMahons creeks) as a proxy for population size, and the age of the barrier (weir_age) as a proxy for the number of generations since isolation of above-barrier populations. We also included the pairwise interactions weir_side:catchment_type and weir_side:weir_age. Locus identity was included as a random intercept. Models were validated a posteriori by checking plots of residuals. Significance of fixed effects was assessed with analysis-of-deviance tables (function Anova in the R package *car*). Post-hoc comparisons of mean allelic richness between populations above and below barriers (pooled into small streams and large rivers separately) was performed using the “*lsmeans*” function implemented in the *lsmeans* package (Lenth, 2016) for R 3.1.3.

To approximate inbreeding due to small *N*_e_, heterozygosity can be scaled by the heterozygosity of a known outbred population using the effective inbreeding coefficient “*F*_e_” that is, *F*_e_ = 1−*H*_e inbred_/*H*_e outbred_, where *H*_e inbred_ is heterozygosity (for neutral variation) of a population in question and *H*_e outbred_ is heterozygosity of an outbred population (Frankham, 1998). We assumed that the pooled samples below each barrier in each stream are not impacted by isolation, and thus can be used as the outbred reference for above-barrier sites in the respective stream. At *F*_e_ of >0.2, inbreeding depression is typically observed for populations of naturally outcrossing species (Frankham, 1995; Woodworth et al., 2002; Szulkin and Sheldon, 2007; Walling et al., 2011), and major reductions in lifetime reproductive success can occur even below *F*_e_ of 0.1 (Huisman et al., 2016).

### Testing for bottlenecks and estimating effective population size (*N*_e_)

To test for evidence of recent reductions in effective population size that might relate to disturbance events, we used BOTTLENECK (Cornuet and Luikart, 1996) on the pooled samples above or below each barrier within each stream. BOTTLENECK performs a test of heterozygosity excess that compares observed results to theoretical expectations based on a population at equilibrium. Tests were performed using the stepwise mutation model (SMM) and the two-phase model of mutation (TPM), although TPM is likely to be more suitable for most microsatellite loci (Cornuet and Luikart, 1996). Default settings considered appropriate for most microsatellites were applied that is, variance for TPM 30, with 70% of mutations following SMM. Wilcoxon’s signed-rank test was applied to determine significance of a heterozygosity excess based on 1,000 iterations (Cornuet and Luikart, 1996).

Effective population sizes (*N*_e_) were estimated using the linkage disequilibrium method (LDNe) (Waples and Do, 2008) in NeESTIMATOR V2.0 (Do et al., 2014). *N*_e_ was calculated for the pooled samples, as estimates from small sample sizes are not reliable (Tallmon et al., 2010). A threshold of 2% was applied to remove rare alleles, which have been shown to bias estimates. The LDNe method makes the following important assumptions: (1) loci are selectively neutral and unlinked, (2) populations are closed, and (3) generations are discrete. Due to low dispersal and breeding likely constrained to small local areas, *G. marmoratus* fail to meet the second assumption. Our estimates do not reflect the global (above or below barrier) ‘population’ but reflect the effective size in the sampled area (Neel et al., 2013). In addition, because *G. marmoratus* are iteroparous and have overlapping generations, they also fail to meet the third assumption. Thus, it is possible that *N*_e_ estimates from this study are downwardly biased by more than 50% (Waples et al., 2014). Nevertheless, *N*_e_ estimates provide useful comparisons between streams, and between populations above and below each barrier.

### Assessing the effect of a barrier, accounting for background genetic structure

To evaluate our power to detect an effect of a barrier through time for various *N*_e_ values and using a space-for-time substitution design (in the absence of temporal data, contemporary spatial patternsare observed to infer likely historical or future changes, see for example, Blois et al. 2013), we ran simulations using a generation-by-generation coalescent algorithm that can simulate spatially limited dispersal (and resulting background genetic structure), implemented in IBDsim 2.0 (Leblois et al. 2003, 2009). Consistent with our *G. marmoratus* data set for each stream, simulated data sets had 90 diploid individuals genotyped at 8 independent microsatellite loci. Mutations of the microsatellite loci followed a generalised stepwise mutation model with variance of 0.36 and a maximum range of allelic states of 60. We fixed the mutation rate to 0.0003, which resulted in ranges of expected heterozygosity values similar to those observed in our data. We simulated a single stream as a linear network with 160 nodes and 200 m between two successive nodes, and sampled 15 individuals per node, every 10 nodes, for 9 nodes starting from the 70^th^ node (Supplementary Fig. S4). The barrier was placed between nodes 125 and 126. This spatial model reflected a scenario where the stream was connected to a larger downstream system with a much smaller upstream catchment. Sampled nodes were subsequently pooled to mimic the spatial design of our real data set (Supplementary Fig. S4). We ran simulations for three dispersal scenarios: (1) Panmixia, (2) IBD, and (3) IBD + asymmetric migration. For each dispersal scenario, we ran simulations for three effective densities (*D*_e_): 3, 10 and 30 individuals per node (or 15, 50 and 150 individuals per km of stream) to cover the range of population sizes in each study stream. According to the formula *N*_e_ = *N*_t_ / (1-*F*_ST_) (Wright 1943), where *N*_t_ is the total number of individuals, these densities correspond to *N*_e_ values for above-barrier populations of ~110, 370 and 1100 individuals, respectively. Limited dispersal for the *IBD* scenario was simulated using a geometric dispersal distribution. Dispersal was spatially limited to obtain levels of genetic structure that were compatible with the values observed in the Yarra River sub-catchment (based on IBD regression slopes among sample sites and pairwise *F*_ST_ values among the pooled samples below each barrier). Maximal dispersal distance was set to 40 nodes, the emigration rate (*e*) and geometric distribution parameter (*g*) were set to 0.5/0.95, 0.25/0.9 and 0.15/0.85 for *D*_e_ of 3, 10 and 30 individuals per node, respectively. To evaluate the impact of asymmetric migration across the barrier, we ran an IBD + asymmetric migration scenario with the same set of parameters, while allowing downstream gene flow at a rate of 0.2. To compare simulation results with a more typical scenario where there is no genetic structure along each stream prior to the installation of barriers, we also ran a Panmixia scenario, for which the maximum dispersal distance was set to 160 nodes (the size of the network), the emigration rate (*e*) and the geometric distribution parameter (*g*) were set to 1/1. For each dispersal scenario and effective density, 50 data sets were simulated assuming no barrier and assuming a complete barrier introduced at various number of generations prior to sampling (5, 10, 15, 20, 25, 50 and 100), chosen to incorporate the range of barrier ages observed in our study area (45–120 years or ~9–24 generations).

For 50 data sets simulated with each combination of parameters (dispersal, *D*_e_ and age of the barrier), we calculated allelic richness in pooled sampled nodes above (nodes 130, 141 and 151) and below the barrier (nodes 100, 110 and 120), and IBD regression slopes for the simulations without a barrier using the GENEPOP 4.1 and *adegenet* package in R 3.1.3. Weir and Cockerham’s pairwise *F*_ST_ was calculated using genpop for each locus and globally among (i) pooled sampled nodes 130, 141, and 151 (above the barrier at node 126) and pooled sampled nodes 100, 110 and 120 (below the barrier at node 126), and (ii) among pooled sampled nodes 100, 110 and 120 and 70, 80 and 90 (all below the barrier at node 126). To determine whether pairwise *F*_ST_ values between pooled nodes above and below the barrier were significantly higher than pairwise *F*_ST_ values between pooled nodes below the barrier, we used a paired Wilcoxon test and each locus as replicate unit. We also conducted similar Wilcoxon tests using the observed data, where pairwise *F*_ST_ values between pooled sites above and below each barrier were compared to pairwise *F*_ST_ values between pooled sites below barriers, but connected via the Yarra River mainstem, within each sub-catchment. Finally, to evaluate our power to detect an effect of a barrier with more markers, we ran the same simulations with 20 microsatellite loci and the same characteristics as above.

## Results

### Low levels of genetic variation

Genotypic data revealed low levels of genetic diversity at eight polymorphic microsatellite loci, with the number of alleles per locus ranging from 3 to 14 per population (Supplementary Table S1 and S3 in Supplementary Material). After false discovery rate correction, there was no significant departure from HWE for any sample site, or for the pooled samples above and below each barrier (Table 1, Supplementary Table S1). No pair of loci showed significant LD. Within the pooled samples, observed and expected heterozygosities were between 0.396–0.516 and 0.396–0.507, respectively, and allelic richness ranged from 2.50 to 4.93 (Table 1). At individual sample sites, observed and expected heterozygosities were between 0.266–0.583 and 0.362–0.534, respectively, and allelic richness ranged from 2.12 to 3.52 (Supplementary Table S1).

### Regional and local population genetic structure

**Fig. 2:**
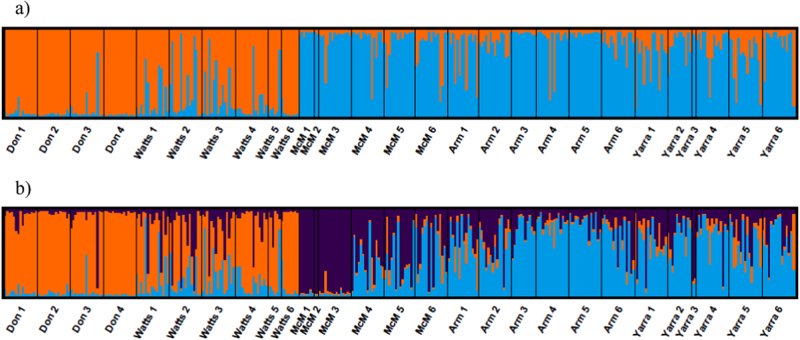
Summary of results of STRUCTURE analysis for *K = *2 **a** and *K = *3 **b**: plots indicate proportional assignment of individuals (bars) to the colour-coded genetic clusters. The population of origin is indicated on the *x* axis in upstream to downstream order for each stream

The most likely value of *K* from the STRUCTURE analysis based on the method of Evanno et al. (2005) was two (Supplementary Table S4 in Supplementary Material), differentiating the two geographically distinct sub-catchments, namely, the Watts River sub-catchment and the upper Yarra River sub-catchment (Fig. 2a). However, LnP(*K*) was highest for *K* = 3 (Supplementary Table S4 in Supplementary Material). With *K* = 3, STRUCTURE identified a third cluster corresponding to samples above the barrier in McMahons Creek (Fig. 2b). Lack of further fine-sale structure within the upper Yarra River sub-catchment was confirmed by a separate analysis of this sub-catchment (Supplementary Appendix S2 in Supplementary Material). Separate analysis of the Watts River sub-catchment revealed a cluster represented predominantly in the above-barrier samples of the Watts River when *K* = 2 was assumed (Supplementary Appendix S2 in Supplementary Material).

**Fig. 3:**
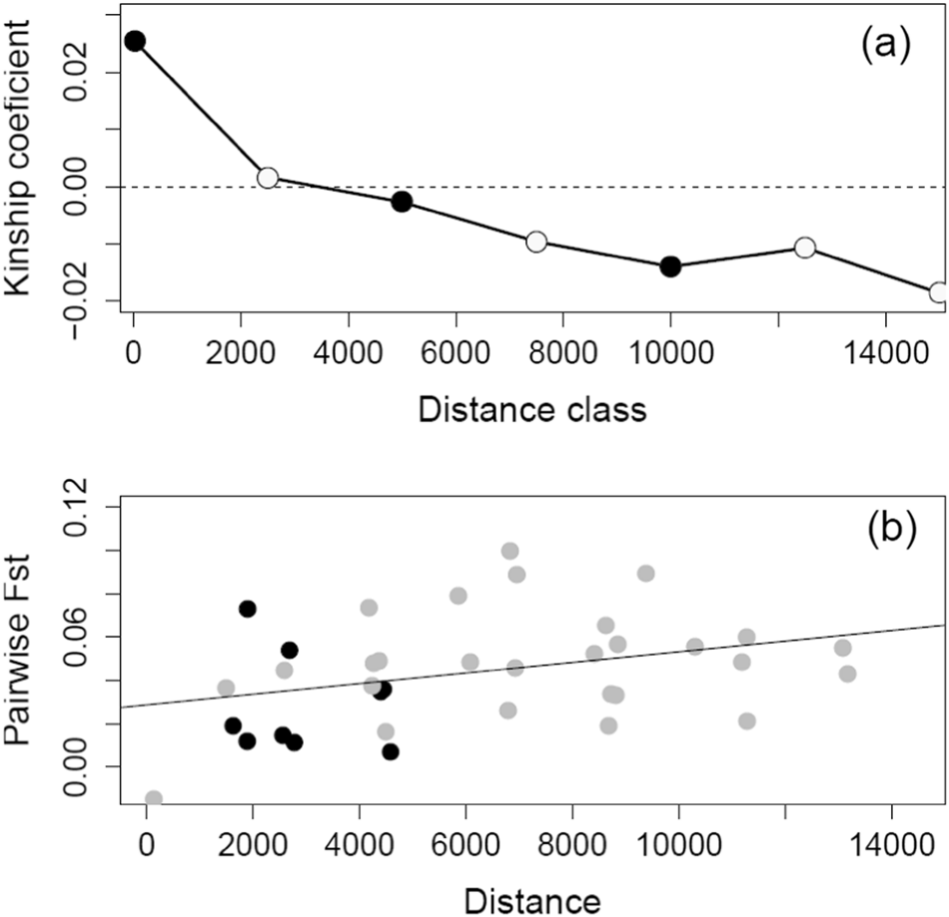
**a** Autocorrelogram showing the Ritland kinship coefficient (Ritland, 1996) as a function of distance (expressed in metres) on pairs of individuals from below-barrier sample sites of the Yarra River sub-catchment. The first distance class represents pairwise comparisons between individuals from the same sampling location. Filled dots indicate departure from the 95% CI for the null hypothesis of a random distribution of genotypes determined by 10,000 random permutations of individuals (first distance class) and individual locations (for the other distance classes). **b** Pairwise *F*_ST_ among sample sites regressed over distance in the Yarra River sub-catchment. Black dots represent pairwise *F*_ST_ values between sample sites above and below barriers in each stream, while grey dots represents pairwise *F*_ST_ values among all nine sites below barriers

Spatial genetic autocorrelation analysis between pairs of individuals below barriers in the Yarra River sub-catchment showed significant and positive kinship values for the first distance class only (Fig. 3a), suggesting limited migration rates in the species. Furthermore, genetic similarity between individuals decreased from 0 to 10 km, suggesting IBD at this scale. A pattern of IBD was suggested by the positive relationship between genetic differentiation and spatial distance among below-barrier sample sites in the Yarra River sub-catchment, although slightly below statistical significance (Fig. 3b, Slope = 2.61 × 10^−6^, 95% CI: (−8.24 × 10^−7^–6.23 × 10^−6^) with distance expressed in metres, Mantel test *P*-value = 0.069).

### Comparing differentiation across barriers to background differentiation

Pairwise *F*_ST_ values between all pooled sites were all significantly different from zero (Table 2). Pairwise *F*_ST_ values between above- and below-barrier pooled samples calculated for each stream (*F*_ST_ = 0.029–0.038) were similar to values between the below-barrier samples pooled within each sub-catchment (*F*_ST_ = 0.023–0.035). The exception was McMahons Creek, where *F*_ST_ between above and below-barrier pooled samples was 0.129.

**Table 2:** *F*_ST_ (lower diagonal) and *D*_est_ (upper diagonal) values for *Gadopsis marmoratus* populations pooled according to stream and either upstream (‘_US_’) or downstream (‘_DS_’) of each water supply weir

Stream	ARM_US_	ARM_DS_	DON_US_	DON_DS_	MCM_US_	MCM_DS_	WAT_US_	WAT_DS_	YAR_US_	YAR_DS_
ARM_US_		0.032	0.115	0.115	0.148	0.027	0.062	0.065	0.038	0.023
ARM_DS_	0.037		0.126	0.153	0.165	0.031	0.065	0.082	0.029	0.018
DON_US_	0.115	0.130		0.029	0.151	0.087	0.056	0.073	0.083	0.088
DON_DS_	0.111	0.147	0.029		0.155	0.109	0.057	0.034	0.128	0.103
MCM_US_	0.162	0.183	0.161	0.157		0.117	0.153	0.193	0.127	0.144
MCM_DS_	0.030	0.035	0.088	0.103	0.129		0.060	0.079	0.017	0.026
WAT_US_	0.062	0.067	0.054	0.053	0.151	0.059		0.034	0.058	0.044
WAT_DS_	0.071	0.091	0.076	0.035	0.203	0.083	0.035		0.092	0.056
YAR_US_	0.042	0.034	0.085	0.119	0.143	0.018	0.057	0.096		0.031
YAR_DS_	0.028	0.023	0.099	0.109	0.172	0.031	0.049	0.067	0.038	

All pairwise *F*_ST_ and *D*_est_ estimates are significant after adjustment for multiple comparisons.

ARM Armstrong Creek, DON Donnellys Creek, MCM McMahons Creek, WAT Watts River, YAR Yarra River

### Effect of barriers on genetic diversity and inbreeding

Linear mixed modelling did not identify a significant effect of weir_side on allelic richness across all sample sites (*P*-value = 0.086), although allelic richness in above-barrier sample sites was significantly lower in smaller catchments (Donnellys, Armstrong and McMahons creeks) compared to larger catchments (Watts and Yarra rivers)—that is, there was a significant weir_side:catchment_type interaction; estimate = −0.62 SE 0.23, *P*-value = 0.007 (Table 3). In smaller catchments, below-barrier sample sites had significantly higher allelic richness than above-barrier ones (estimate = 0.314 SE 0.149, *P*-value = 0.037), while in larger catchments, there was no significant difference (estimate = -0.308 SE 0.175, *P*-value = 0.08). There was no effect of *barrier_age* or interaction with catchment_type on allelic richness. The variance estimate for the random intercept on loci was 1.69 (SD 1.3).

**Table 3:** Linear mixed model results to test the direct effects and interaction of barriers (*weir_side*), catchment size (*catchment_type*) and barrier age (*weir_age*) on *Gadopsis marmoratus* populations in the Yarra River system

Explanatory variable	Chisq	Df	*P*-value
*Fixed effect*			
Weir_side	2.935	1	0.086
Catchment_type	0.125	1	0.723
Weir_age	0.001	1	0.982
*Interactions*			
Weir_side: Catchment_type	7.259	1	**0.007**
Weir_side: Weir_age	0.584	1	0.444

Significant *P*-value is shown in bold

The effective inbreeding coefficient (*F*_e_) calculated in pooled samples above barriers within each stream was positive in Donnelly’s Creek (*F*_e_ = 0.055) and McMahons Creek (*F*_e_ = 0.165, Table 1), suggesting inbreeding in *G. marmoratus* populations within these streams, particularly McMahons Creek.

### Bottleneck tests and estimation of N_e_

BOTTLENECK test results provided evidence for a recent bottleneck in the pooled samples above McMahons Creek weir under the TPM and SMM mutation models, but no bottlenecks were found in any other population (Table 4). Confidence intervals for *N*_e_ estimates included infinite values for populations in the Watts and Yarra rivers and in Armstrong Creek, suggesting insufficient power in our data to estimate *N*_e_ in these three larger streams (Tallmon et al. 2010). For the pooled samples above and below barriers within the two smallest creeks, small *N*_e_ estimates were obtained in Donnellys Creek and were similar above and below the barrier (10.9 and 10.7, respectively), while in McMahons Creek, *N*_e_ was lower above than below the barrier (8.3 and 30.8, respectively).

**Table 4:** BOTTLENECK results for *Gadopsis marmoratus* populations upstream and downstream of water supply barriers in the Yarra River system^a,b^

Waterway	Upstream or Downstream of Weir	TPM	SMM	Mode shift
Armstrong	Upstream	0.770	0.980	No
	Downstream	0.656	0.961	No
Donnellys	Upstream	0.230	0.629	No
	Downstream	0.234	0.711	No
McMahons	Upstream	**0.020**	**0.039**	Yes
	Downstream	0.055	0.406	No
Watts	Upstream	0.371	0.844	No
	Downstream	0.844	0.990	No
Yarra	Upstream	0.594	0.852	No
	Downstream	0.963	0.990	No

^a^Probability of heterozygote excess according to the Wilcoxon sign-rank test under the TPM (Two-phased model of mutation) and SMM (Stepwise Mutation Model) for each population.

^b^Significant *P*-values are shown in bold.

### Assessing the effect of a barrier, accounting for background genetic structure

With limited dispersal (IBD and IBD + asymmetric migration scenarios) and for each effective density, simulated data sets without a barrier yielded pairwise *F*_ST_ values among pooled sampled nodes above and below node 126 (barrier location) and IBD regression slopes among sampled nodes consistent with observed values between sample sites below barriers in the Yarra River sub-catchment (Table 5). Simulations with 20 loci indicated increased power to detect sigificant IBD patterns compared to 8 loci, where using the 20 loci, simulation IBD regression slopes ranged from 3.026 × 10^−6^ (SD 1.992 × 10^−6^) to 3.275 × 10^−6^ (SD 1.587 × 10^−6^) and the percentage of significant Mantel tests ranged from 66 to 96. This result compares to the simulations with eight loci where regression slopes ranged from 2.395 × 10^−6^ (SD 3.431 × 10^−6^) to 3.417 × 10^−6^ (SD 2.562 × 10^−6^) and the percentage of significant Mantel tests ranged from 28 to 76. Even without a barrier, allelic richness in pooled upstream nodes was slightly lower than in pooled downstream nodes (Supplementary Table S8).

**Table 5:** IBDsim simulation parameter summary and mean values based on 50 simulations without a barrier, compared to the observed data set for all sites downstream of barriers in the Yarra sub-catchment

		Dispersal scenario					
	Observed data set	Panmixia			IBD		
Density of individuals per km (*D*_e_)		15	50	150	15	50	150
Dispersal parameters							
maximum dispersal distance		160 nodes			40 nodes		
emigration rate (e)		1	1	1	0.50	0.25	0.15
geometric distribution (g)		1	1	1	0.95	0.90	0.85
Summary statistics							
IBD slope (SD)	2.61 × 10^−6^	NA	NA	NA	2.395 × 10^−6^ (3.431 × 10^−6^)	3.417 × 10^−6^ (2.562 × 10^−6^)	3.306 × 10^−6^ (1.991 × 10^−6^)
*F*_ST_ above-below node 125 (SD)	0.023–0.035	0 (0)	0 (0)	0 (0)	0.027 (0.023)	0.029 (0.011)	0.027 (0.009)
*A*_*r*_ ratio (pooled nodes 100,110,120)/pooled nodes 130,140,150)		1.000	1.000	1.000	0.951	0.957	0.948
% IBD mantel tests significant					28	56	76

**Fig. 4:**
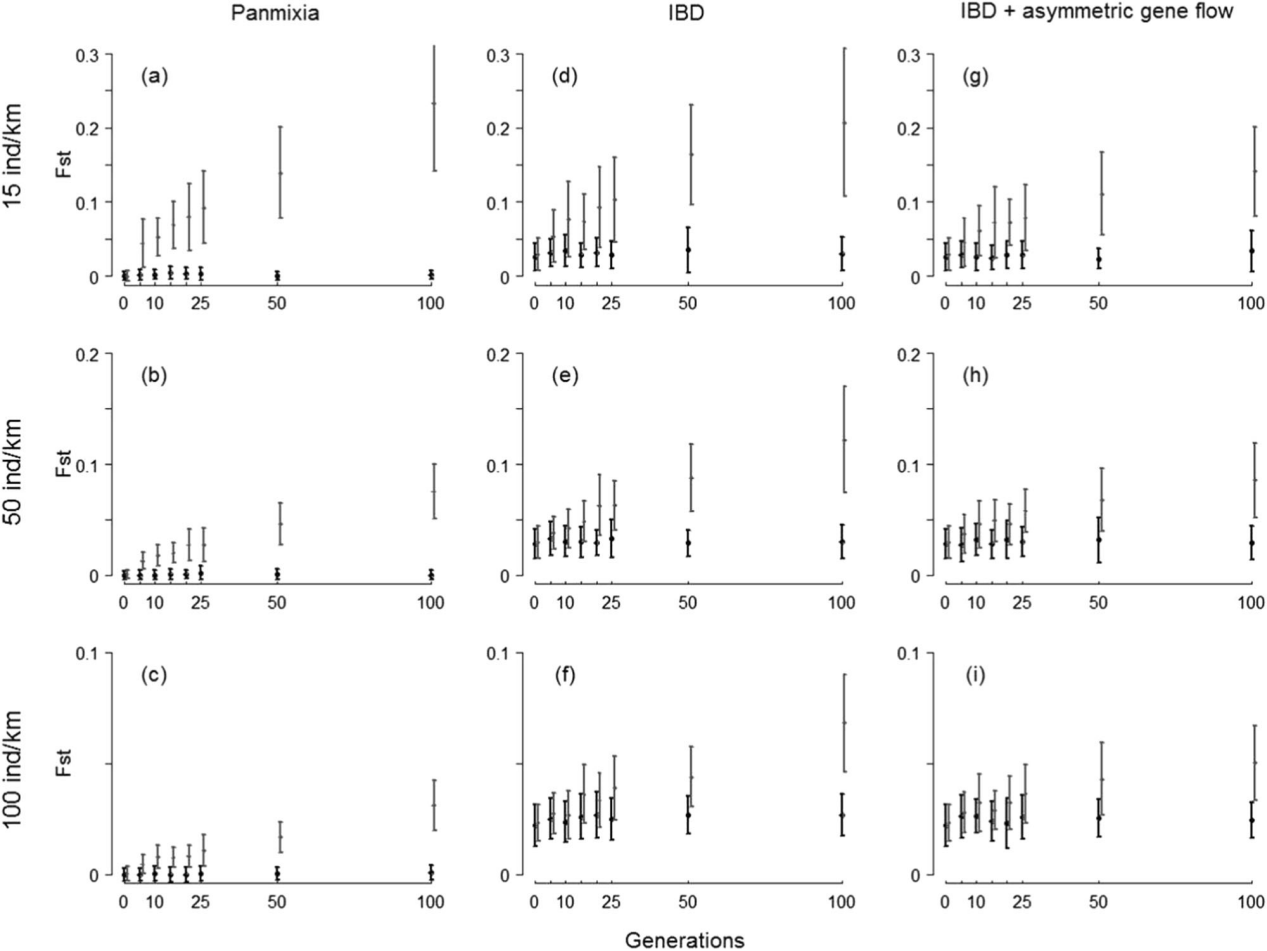
Simulated increases in population differentiation (pairwise *F*_ST_ ± SD based on 50 simulated data sets) with time (number of generations) using eight microsatellites markers. Pairwise *F*_ST_ values are shown (i) between pooled nodes above the barrier (nodes 130, 140 and 150) and pooled nodes below the barrier (nodes 100, 110, 120) (grey), and (ii) among two pools of nodes below the barrier (nodes 100, 110, 120 compared to nodes 100, 110, 120) (black) per dispersal scenario: Panmixia **a**–**c**, IBD **d**–**f** and IBD + asymmetric migration **g**–**i** and effective density: 15 individuals per km **a**, **d**, **g**, 50 individuals per km **b**, **e**, **h** and 150 individuals per km **c**, **f**, **i**

Simulations with a complete barrier suggested that low effective densities (*D*_e_) would be required for genetic drift to increase *F*_ST_ values substantially (for example, *F*_ST_>0.1) over the number of generations since the installation of barriers in the study area (up to ~25 generations) regardless of the dispersal scenario (Fig. 4). However, substantial differences in the ability to detect a barrier effect with a space-for-time substitution design were evident between Panmixia and IBD scenarios (Supplementary Table S8). In the Panmixia scenario, within only 15 generations, significantly higher *F*_ST_ values were detected across the barrier in more than half the simulated data sets with low and medium effective densities (15 and 50 individuals per km), while 100 generations are likely to be required in the IBD scenario to reach a similar threshold (Fig. 4). Simulations with asymmetric migration showed that downstream gene flow slightly delays the development of genetic differentiation and the ability to detect a barrier effect (Fig. 4 and Supplementary Table S8). Allelic richness in pooled nodes above the barrier was predicted to progressively decrease with the number of generations since barrier installation.

Variation in *F*_ST_ estimates was high among our simulated data sets, and most likely reflects the limited statistical power of eight polymorphic loci. Indeed, simulations with 20 microsatellite loci with same characteristics showed a substantial increase in power to detect barrier impacts (Supplementary Table S9 and Supplementary Fig. S5 in Supplementary Material).

Comparing these simulations with observed pairwise *F*_ST_ values above and below each barrier compared to pooled sites below barriers (Supplementary Table S10) and the number of generations since barrier installation for each stream, the above-barrier population of McMahons Creek is likely to have recently experienced strong genetic drift–with effective densities much lower than 15 individuals per km (global upstream population of <110 individuals). This is consistent with our *N*_e_ estimate (~10 individuals over the sampled area) and the detection of a bottleneck. For Donnellys Creek, however, where the barrier is the oldest (~25 generations) and *N*_e_ was estimated to be similar to McMahons Creek above the barrier, the low level of differentiation (*F*_ST_ = 0.029) cannot be attributed to downstream gene flow only.

## Discussion

### Evaluating the impacts of barriers on low-mobility species

We sought to address a lack of studies that assess the genetic impacts of artificial barriers on freshwater fish species with low mobility. On the basis of spatially explicit simulations and an extensive empirical data set, our study highlights the importance of accounting for background genetic structure in species of low mobility to avoid falsely attributing genetic impacts to barriers. For example, a simple test of genetic differentiation across a barrier may be insufficient to establish the effect of a barrier alone. In these cases, barrier effects can only be identified if there is a significant increase in genetic differentiation that exceeds background levels of genetic structure. This can be assessed with temporal sampling (Schwartz et al., 2007) or with a space-for-time substitution study design (such as the present study). Genetic impacts from barriers were most evident in one of our small streams. This result is likely to be associated with a recent bottleneck and restricted gene flow imposed by the barrier that limits population recovery. In the other study streams, a lack of evidence for significant barrier effects can be attributed to larger population sizes or, in the case of the smallest barrier, gene flow across the barrier.

### Background levels of genetic structure and diversity

Genetic differentiation was greatest between the two geographically distinct sub-catchments: the ‘Upper Yarra River sub-catchment’ (Upper Yarra River, Armstrong Creek and McMahons Creek) and the ‘Watts River sub-catchment’ (Watts River and Donnellys Creek). Given that the two sub-catchments are currently hydrologically connected via the mainstem of the Yarra River, differentiation between these two sub-catchments is likely due to the low mobility of *G. marmoratus* (Koehn, 1986; Khan et al., 2004; Koster and Crook, 2008). Within the Yarra River sub-catchment, a pattern of isolation-by-distance was suggested and spatial genetic autocorrelation was significant between individuals at the scale of individual sample sites only. Inference of poor dispersal is consistent with the findings of substantial genetic structure and population differentiation at spatial scales of <5 km to 100’s of km between study sites for northern *G. marmoratus* within the Murray-Darling Basin (Huey et al., 2017; Lean et al., 2017). While genetic diversity of *G. marmoratus* within the study area was generally low at all sample sites, low genetic diversity was also found within other populations across the broader range of the species complex (Huey et al., 2017; Lean et al., 2017). A strong effect of drift due to restricted migration rates and low population effective size is the most likely explanation and has been suggested to relate either to fragmentation due to human activities or to life history characteristics of the species (Huey et al., 2017; Arias et al., 2013; Lean et al., 2017).

### Effects of barriers on population differentiation and genetic diversity in structured populations

Theory and simulation-based studies predict that the effect of genetic drift on genetic diversity and structure depends on the effective population size and the number of generations since population isolation (Leblois et al., 2006; Gauffre et al., 2008; Landguth et al., 2010). Failing to account for prior population structure at small spatial scales (for example, within streams) for low-mobility species, however, could lead to erroneously attributing natural levels of differentiation to the effect of a barrier; but examples where local background structure is present and explicitly accounted for in tests of barrier effects in freshwater systems appear to be rare. The presence of population structure also results in longer lag times before genetic patterns can be detected (Leblois et al., 2006; Landguth et al., 2010). Our spatially explicit simulations suggest that, even without barriers, we could expect: (i) slightly lower genetic diversity (*A*_r_) within upper reaches compared to the lower reaches, and (ii) significant differentiation between upper and lower reaches within our study streams. Hence, in non-panmictic populations such as *G. marmoratus* in our study area, significant genetic differentiation (*F*_ST_) across barriers cannot be solely attributed to barrier effects.

Our simulations also showed that, when a barrier was introduced, if the number of generations since population isolation is low (<25 generations), substantial loss of diversity above a barrier and development of genetic differentiation across barriers will only occur in IBD populations when effective densities are small (for example, 15 individuals per km). In addition, when asymmetrical gene flow was taken into account in IBD populations, effects of barriers were unlikely to become evident even in the smallest simulated effective density of 15 individuals per km for up to 50 generations. Results from the IBD simulations with and without asymmetrical gene flow were in clear contrast to those for panmictic populations. In the simulations for panmictic populations, substantial loss of diversity above a barrier and development of genetic differentiation across barriers can be expected within much smaller timeframes (for example, <5 generations for effective densities of up to 50 individuals per km) and for larger population densities (for example, <25 generations for effective densities up to 150 individuals per km). On the basis of our estimates of effective population sizes and number of generations since the presence of barriers (~9–24), an effect of barriers on genetic structure and diversity was only expected in the two smallest study streams (Donnellys and McMahons creeks).

High variance among simulated data sets reflects the limited ability of a small number of microsatellite markers with low diversity to detect genetic effects (Landguth et al., 2012). This inference was supported results from our simulations using 20 loci, which suggest that impacts from barriers had the potential to be detected much sooner with a greater number of loci (for example, for IBD scenarios with or without asymmetrical gene flow, within 5–10 generations for effective densities of 15 individuals per km or within 15–50 generations for effective densities of 50 and 100 individuals per km, respectively). Simulations with 20 loci also indicated an increased power to detect IBD compared to eight loci based on the percentage of simulations with a significant Mantel test.

We originally hypothesised that populations isolated upstream of barriers would have lower genetic diversity compared to downstream populations that are more interconnected over a much larger area of habitat. In particular, we hypothesised that genetic diversity in isolated populations upstream of barriers would vary depending on barrier age, the size of the upstream population and disturbance history. Using our observed data set, we found support for the effect of population size, but not the age of the barrier. The size of the populations (based on catchment area) was found to be important for retention of genetic diversity above barriers. Importantly, we cannot relate this result directly to barrier effects, because simulations showed that even in the absence of a barrier upstream, genetic diversity is still expected to be lower above barriers. In addition, contrary to expectations based on simulations, we found more genetic diversity above barriers in the two largest streams (upper Yarra and Watts rivers)–indicating likely large densities of individuals above barriers in these two rivers. This finding is consistent with those of Whiteley et al. (2010, 2013), who observed in brook trout (*Salvelinus fontinalis*) and cutthroat trout (*Oncorhynchus clarkii clarkii*) populations that the largest patches above barriers had higher genetic diversity than adjacent below-barrier patches, and Gouskov et al. (2016), who observed that large lake populations of European chub (*Squalius cephalus*) were important for sustaining genetic diversity in fragmented rivers. It is likely that large reservoirs in our system (Maroondah reservoir for the Watts River and upper Yarra reservoir for the Yarra River) sustain large populations and provide refuge habitats for *G. marmoratus* during droughts, such as those experienced in 1982–83 and late 1996–mid 2010.

On the other hand, genetic differentiation across the barrier in McMahons Creek was the highest and was associated with a reduction in genetic diversity above the barrier. This suggests that low diversity is likely to be the driver of the differentiation (similar to the results of Coleman et al. 2013 for dwarf galaxias (*Galaxiella pusilla*) and Weeks et al. 2016 for Australian mammals). Compared to expectations based on simulations, the population density in McMahons Creek is anticipated to be below the lowest simulated effective density (<15 individuals per km), which is consistent with evidence of a recent bottleneck in the upper McMahons Creek population. One likely explanation for a bottleneck in McMahons Creek is the 1983 wildfires that affected the entire McMahons Creek catchment (Woodgate 1984). Impacts of fire on aquatic ecosystems include a range of chemical, physical and biological changes that can be direct and immediate, as well as indirect and long-term. These impacts are strongest for populations isolated by anthropogenic activities, where fish are unable to recover post-fire via immigration from below into affected areas (Gresswell, 1999; Lyon and O’Connor, 2008). For example, habitat connectivity was inferred to be important for the recovery of rainbow trout (*Oncorhynchus mykiss*) populations following catchment disturbance from fires, where genetic diversity was lower in populations upstream of barriers due to culverts (Neville et al., 2009). By preventing gene flow following a bottleneck, the water supply weir on McMahons Creek appears to have impeded recovery of the upstream population compared to the downstream population that has greater genetic diversity.

Given that the Donnellys Creek population above the barrier has the smallest catchment and the oldest weir, we expected loss of genetic diversity and elevated pairwise *F*_ST_ above and below the barrier. Whilst loss of genetic diversity upstream was lower than expected, *F*_ST_ was smaller than predicted in simulations, even with substantial downstream gene flow. This finding suggests that downstream gene flow alone cannot explain results for this stream. Donnellys Creek weir is the lowest of the study barriers (~1.5 m high), so it is plausible that there has also been some upstream migration of individuals over the weir during extreme stream flows or from undocumented translocations into the upstream weir pool. Homogenisation of gene pools of Roanoke logperch (*Percina rex*) above and below older (c. 1920), but smaller artificial barriers (~10 m high), was thought to be associated with at least one-way gene flow across the barrier (Roberts et al., 2013).

### Management implications

In the context of the classic Wright-Fisher model for closed panmictic populations, the minimum effective population sizes necessary for viable isolated populations are estimated to be ~100 individuals to avoid inbreeding depression in the short-term, and 1000 or more to maintain adaptive potential in the long-term (Frankham et al., 2014). In IBD populations, however, where dispersal is restricted and there is a higher probability that individuals preferentially breed with those in close proximity, detection of genetic impacts such as loss of genetic diversity due to habitat reduction, is expected to differ from panmictic populations for a given population size and temporal scale (Leblois et al., 2006). Accordingly, in order to accurately evaluate the impacts of barriers on species with low mobility and determine the need for management intervention, it is essential to take underlying IBD genetic patterns into account.

Observations of genetic impacts in this study were not related to barrier age and, instead, we demonstrated impacts in one stream within 45 years of isolation (approximately nine generations). The *G. marmoratus* population above the McMahons Creek weir is likely to be at risk from inbreeding and poor adaptive potential, which is supported by a higher effective inbreeding coefficient (*F*_e_ = 0.161). This finding highlights the importance of maintaining population connectivity in small streams where genetic impacts of a barrier due to restricted gene flow following a disturbance event can develop within just a few generations. Immediate actions to improve connectivity and gene flow could include the installation of fishways on the smaller streams showing signs of genetic drift above barriers, as demonstrated for European chub (Gouskov et al., 2016). Where a fishway is not feasible (for example, due to site or cost constraints), intermittent translocations from downstream populations might be advantageous for improving genetic diversity and adaptive potential–especially in upper McMahons Creek, where a recent bottleneck leading to high inbreeding and loss of genetic diversity was detected.

This study indicates that activities aiming to facilitate gene flow for freshwater fish along streams are particularly relevant for small, isolated populations that are more vulnerable to disturbance events (such as wildfire or drought) and are currently unable to recover via gene flow from downstream populations. In addition, the need for assisted gene flow is increased for species with low mobility that may take longer to recover from genetic impacts than more mobile species (Landguth et al., 2010). Loss of genetic diversity in these situations must be addressed alongside more commonly considered threats, in this case exemplified by habitat modification (especially removal of large woody debris), sedimentation (for example, smothering of eggs), altered stream flows, interactions with alien species (such as brown trout, *Salmo trutta*) and recreational fishing (Koehn and O’Connor, 1990; Lintermans, 2007).

To determine the effectiveness of interventions to increase gene flow, genetic monitoring is recommended, that is, collection of tissue samples from populations upstream and downstream of (former) barriers to assess temporal changes in *N*_*e*_, *A*_r_, or *H*_e_ (see Category II monitoring described in Schwartz et al., 2007). As part of further genetic monitoring and evaluation of the need for genetic intervention in the larger streams, the application of a greater number of markers would be more powerful for early detection of genetic impacts.

## Data archiving

Data available from the Dryad Digital Repository: http://dx.doi.org/10.5061/dryad.hn050.

## Electronic supplementary material

Supplemetal material
