# Supplementary material for: Artificial barriers prevent genetic recovery of small isolated populations of a low-mobility freshwater fish
Source: Heredity (Edinb). 2018 Jan 12;120(6):515–32. doi: 10.1038/s41437-017-0008-3 (PMC5943333; doi:10.1038/s41437-017-0008-3)
Supplement: Supplementary file 1 — Supplemetal material [file 41437_2017_8_MOESM1_ESM.docx]

**Supplementary Material**

**Appendix S1.** PCR conditions for 11 microsatellite DNA markers developed for *Gadopsis marmoratus* (Ling *et al*., 2013) and amplified in two separate multiplex reactions (Plex A and B) following Beheregaray *et al*. (2004).

Plex A (Gama01, Gama02, Gama03, Gama07, Gama08, Gama10 and Gama11) was amplified with denaturation at 94⁰C for 3 minutes, four touchdown cycles of 94⁰C for 20 seconds, 63⁰C for 45 seconds (decrease by 2⁰C at each cycle until 57⁰C) and 72⁰C for 1 minute, followed by 35 cycles of these same conditions but annealing at 55⁰C, with extension at 72⁰C for 4 minutes. Plex B (Gama04, Gama05 Gama06 and Gama12) was amplified with denaturation at 94⁰C for 2 minutes, six touchdown cycles of 94⁰C for 20 seconds, 62⁰C for 30 seconds (decrease by 2⁰C at each cycle until 52⁰C) and 72⁰C for 3 minutes followed by 35 cycles at the same conditions but annealing at 55⁰C with extension at 72⁰C for 10 minutes. The PCR aliquot contained 10 ng/ul of DNA template, 1x Mango Taq Reaction Buffer (Bioline, London, UK), 0.2 mM of dNTPs (Sigma-Aldrich, St. Louis, USA), 3mM of MgCl2, 0.05 – 0.4 µM of primers, 0.1 mg/ml of BSA (New England Biolabs, Ipswich, USA), 0.05 U of Mango Taq DNA polymerase (Bioline, London, UK), and sterile water up to the final reaction volume. Reaction volumes were 10 µl and 5 µl for Plex A and B, respectively.

**Table S1.** Number of individuals (*n*), geographic coordinates, average number of allele per locus (*N*_a_), allelic richness (*A*_r_) corrected for a minimal sample size of 11 individuals, expected (*H*_e_) and observed heterozygosity (*H*_o_), *P*-value of the Hardy-Weinberg Equilibrium (HWE) exact test and F_IS_ value calculated for each site.

| **Waterway** | **Upstream or Downstream of Weir** | **Site** | ***n*** | **Latitude** | **Longitude** | ***N*_a_** | ***A*_r_**  **(*n*=6)** | ***H*_o_** | ***H*_e_** | **HWE**  ***P*-value** | ***F*_IS_** |
| --- | --- | --- | --- | --- | --- | --- | --- | --- | --- | --- | --- |
| Armstrong | Upstream | ARM01 | 14 | -37°35'51.44" | 145°52'8.75" | 3.63 | 2.78 | 0.432 | 0.451 | 0.142 | 0.040 |
|  |  | ARM02 | 15 | -37°36'42.56" | 145°52'17.85" | 3.63 | 2.98 | 0.525 | 0.464 | 0.994 | -0.132 |
|  |  | ARM03 | 11 | -37°38'9.67" | 145°51'36.22" | 3.38 | 2.79 | 0.466 | 0.419 | 0.574 | -0.113 |
|  | Downstream | ARM04 | 15 | -37°38'20.8" | 145°51'35.56" | 3.75 | 2.95 | 0.500 | 0.449 | 0.541 | -0.113 |
|  |  | ARM05 | 15 | -37°39'38.43" | 145°51'4.78" | 3.88 | 3.03 | 0.433 | 0.433 | 0.806 | -0.001 |
|  |  | ARM06 | 15 | -37°40'22.14" | 145°50'58.23" | 3.88 | 2.95 | 0.467 | 0.424 | 0.028 | -0.100 |
| Donnellys | Upstream | DON01 | 15 | -37°36'55.33" | 145°32'0.44" | 3.50 | 3.05 | 0.583 | 0.534 | 0.824 | -0.092 |
|  |  | DON02 | 15 | -37°37'38.25" | 145°32'8.16" | 2.50 | 2.33 | 0.450 | 0.385 | 0.037 | -0.168 |
|  | Downstream | DON03 | 15 | -37°37'42.83" | 145°32'7.95" | 3.38 | 2.84 | 0.525 | 0.476 | 0.360 | -0.104 |
|  |  | DON04 | 15 | -37°38'11.24" | 145°32'6.98" | 3.50 | 2.92 | 0.558 | 0.505 | 0.935 | -0.105 |
| McMahons | Upstream | MCM01 | 7 | -37°44'20.23" | 145°54'8.09" | 2.13 | 2.12 | 0.357 | 0.421 | 0.900 | 0.152 |
|  |  | MCM02 | 2 | -37°43'39.77" | 145°53'37.76" | 1.88 | NA | - | - | - | - |
|  |  | MCM03 | 15 | -37°43'10.22" | 145°53'0.09" | 2.25 | 2.21 | 0.425 | 0.362 | 0.473 | -0.172 |
|  | Downstream | MCM04 | 15 | -37°42'53.47" | 145°52'31.99" | 3.38 | 2.83 | 0.492 | 0.461 | 0.913 | -0.066 |
|  |  | MCM05 | 14 | -37°42'41.62" | 145°51'20.94" | 3.88 | 3.11 | 0.518 | 0.478 | 0.411 | -0.083 |
|  |  | MCM06 | 15 | -37°42'4.79" | 145°49'57.88" | 3.25 | 2.8 | 0.408 | 0.456 | 0.619 | 0.105 |
| Watts | Upstream | WAT01 | 15 | -37°36'48.43" | 145°37'30.5" | 3.88 | 3.27 | 0.550 | 0.508 | 0.112 | -0.083 |
|  |  | WAT02 | 15 | -37°36'57.08" | 145°36'11.46" | 4.38 | 3.36 | 0.500 | 0.478 | 1.000 | -0.045 |
|  |  | WAT03 | 15 | -37°37'28.74" | 145°34'52.33" | 4.50 | 3.52 | 0.471 | 0.521 | 0.292 | 0.097 |
|  | Downstream | WAT04 | 15 | -37°38'37.65" | 145°32'52.04" | 4.38 | 3.18 | 0.475 | 0.491 | 0.111 | 0.033 |
|  |  | WAT05 | 6 | -37°38'24.04" | 145°32'8.65" | 3.13 | 3.13 | 0.375 | 0.477 | 0.233 | 0.214 |
|  |  | WAT06 | 8 | -37°39'5.47" | 145°30'58.38" | 2.75 | 2.56 | 0.266 | 0.368 | 0.038 | 0.279 |
| Yarra | Upstream | YAR01 | 15 | -37°43'45.71" | 146°2'47.66" | 4.13 | 3.1 | 0.508 | 0.490 | 0.730 | -0.037 |
|  |  | YAR02 | 11 | -37°44'4.25" | 146°0'58.38" | 3.13 | 2.74 | 0.443 | 0.437 | 0.934 | -0.013 |
|  |  | YAR03 | 2 | -37°43'31.93" | 146°0'15.1" | 1.88 | NA | - | - | - | - |
|  | Downstream | YAR04 | 15 | -37°40'8.53" | 145°53'35.84" | 3.88 | 3.02 | 0.442 | 0.434 | 1.000 | -0.017 |
|  |  | YAR05 | 15 | -37°40'24.85" | 145°52'27.58" | 3.00 | 2.46 | 0.358 | 0.381 | 0.973 | 0.059 |
|  |  | YAR06 | 15 | -37°40'21.98" | 145°50'58.72" | 3.75 | 2.9 | 0.408 | 0.403 | 0.330 | -0.013 |

**Table S2.** Summary length and weight information for *Gadopsis marmoratus* captured from the study sites. Juveniles <60mm were removed from genetic analyses.

| **Waterway** | **Upstream or Downstream of Weir** | *n* | **Total length (mm)** | | | | **Weight (g)** | | | |
| --- | --- | --- | --- | --- | --- | --- | --- | --- | --- | --- |
|  |  |  | min | max | mean | SE | Min | max | mean | SE |
| Armstrong | Upstream | 45 | 38 | 355 | 158.4 | 13.7 | <1 | 382 | 76.3 | 15.3 |
|  | Downstream | 45 | 77 | 422 | 185.8 | 14.5 | 2 | 582 | 112.1 | 21.4 |
| Donnellys | Upstream | 30 | 162 | 321 | 248.7 | 8.2 | 42 | 284 | 156.3 | 14.0 |
|  | Downstream | 30 | 122 | 374 | 235.7 | 12.8 | 18 | 504 | 150.3 | 21.4 |
| McMahons | Upstream | 24 | 118 | 395 | 274.1 | 14.0 | 16 | 478 | 200.9 | 25.5 |
|  | Downstream | 45 | 49 | 410 | 241.5 | 11.5 | <1 | 536 | 161.3 | 19.8 |
| Watts | Upstream | 45 | 73 | 358 | 166.4 | 11.4 | 4 | 462 | 76.4 | 16.3 |
|  | Downstream | 29 | 110 | 455 | 275.7 | 18.0 | 10 | 830 | 237.1 | 42.7 |
| Yarra | Upstream | 28 | 106 | 351 | 173.8 | 12.3 | 12 | 438 | 76.9 | 19.5 |
|  | Downstream | 45 | 67 | 406 | 255.9 | 12.6 | 4 | 584 | 192.9 | 20.8 |

**Table S3.** *Gadopsis marmoratus* microsatellite locus summary.

| **Locus** | **Total Alleles** | **Size Range (bp)** |
| --- | --- | --- |
| Gama01 | 14 | 320-352 |
| Gama02 | 1 | 160 |
| Gama03 | 3 | 155-159 |
| Gama07 | 7 | 220-242 |
| Gama08 | 9 | 259-337 |
| Gama10 | 5 | 152-164 |
| Gama11 | 4 | 203-209 |
| Gama04 | 6 | 135-149 |
| Gama05 | 1 | 234 |
| Gama06 | 14 | 169-199 |
| Gama12 | 1 | 182 |

**Appendix S2.** Hierarchical STRUCTURE analysis across all study sites and within each sub-catchment.

**A) Full dataset**

**Table S4.** STRUCTURE analysis summary for *K* 1–10 performed on the full dataset. The

highest Delta *K* value and the highest mean LnP(K) are in bold font.

| *K* | Reps | Mean LnP(*K*) | St dev LnP(*K*) | Ln'(K) | \|Ln''(K)\| | Delta *K* |
| --- | --- | --- | --- | --- | --- | --- |
| 1 | 10 | -6299.12 | 0.14 | — | — | — |
| **2** | **10** | **-6007.07** | **0.45** | **292.05** | **205.22** | **457.563** |
| **3** | **10** | **-5920.24** | **0.64** | **86.83** | **124.625** | **195.918** |
| 4 | 10 | -5958.04 | 23.73 | -37.795 | 38.77 | 1.634 |
| 5 | 10 | -5957.06 | 29.98 | 0.975 | 134.83 | 4.497 |
| 6 | 10 | -6090.92 | 61.15 | -133.855 | 234.655 | 3.837 |
| 7 | 10 | -6459.43 | 295.42 | -368.51 | 420.65 | 1.424 |
| 8 | 10 | -6407.29 | 167.94 | 52.14 | 440.875 | 2.625 |
| 9 | 10 | -6796.02 | 235.79 | -388.735 | 51.425 | 0.218 |
| 10 | 10 | -7133.33 | 156.01 | -337.31 | — | — |

**B.1) Upper Yarra River sub-catchment**

**Table S5.** STRUCTURE analysis summary for *K* 1–10 performed on the Upper Yarra River sub-catchment dataset. The highest Delta *K* value and the highest mean LnP(K) are in bold font.

| *K* | Reps | Mean LnP(*K*) | St dev LnP(*K*) | Ln'(K) | \|Ln''(K)\| | Delta *K* |
| --- | --- | --- | --- | --- | --- | --- |
| 1 | 10 | -3571.9 | 0.11 | — | — | — |
| **2** | **10** | **-3554.72** | **3** | **17.18** | **309.66** | **103.26** |
| 3 | 10 | -3847.2 | 175.02 | -292.48 | 84.91 | 0.48 |
| 4 | 10 | -4054.77 | 110.34 | -207.57 | 170.48 | 1.54 |
| 5 | 10 | -4091.86 | 54.29 | -37.09 | 28.93 | 0.53 |
| 6 | 10 | -4157.88 | 32.16 | -66.02 | 22.52 | 0.70 |
| 7 | 10 | -4201.38 | 89.94 | -43.5 | 7.93 | 0.09 |
| 8 | 10 | -4252.81 | 73.34 | -51.43 | 163.5 | 2.23 |
| 9 | 10 | -4140.74 | 62.89 | 112.07 | 153.75 | 2.44 |
| 10 | 10 | -4182.42 | 57.62 | -41.68 | — | — |

**Figure S1.** Summary of results of STRUCTURE analysis on the Upper Yarra River sub-catchment for *K* = 2 (a) and *K* = 3 (b): plots indicate proportional assignment of individuals (bars) to the colour-coded genetic clusters. The population of origin is indicated on the *x*-axis in upstream to downstream order for each stream.

**
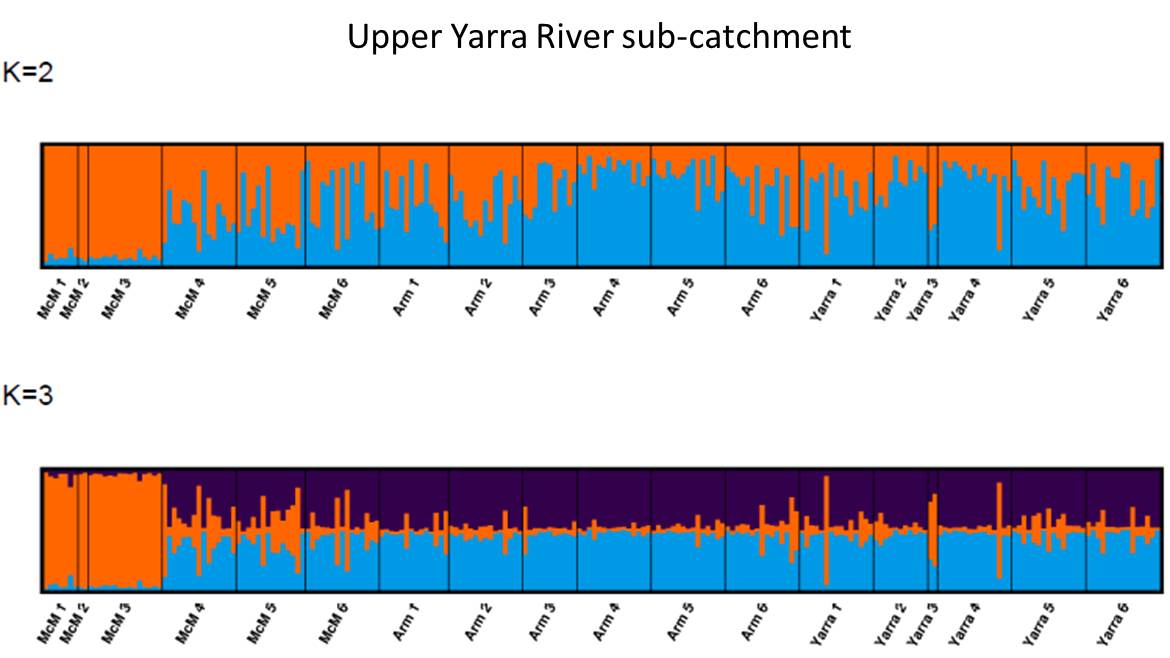
**

**B.2) Upper Yarra River sub-catchment without samples collected upstream of the barrier in McMahons Creek**

**Table S6.** STRUCTURE analysis summary for *K* 1–10 performed on the Upper Yarra River sub-catchment dataset excluding samples collected upstream of the barrier in McMahons Creek. The highest Delta *K* value and the highest mean LnP(K) are in bold font.

| *K* | Reps | Mean LnP(*K*) | St dev LnP(*K*) | Ln'(K) | \|Ln''(K)\| | Delta *K* |
| --- | --- | --- | --- | --- | --- | --- |
| **1** | **10** | **-3142.78** | **0.229976** | **—** | **—** | **—** |
| **2** | **10** | **-3173.02** | **10.32987** | **-30.24** | **29.71** | **2.876125** |
| 3 | 10 | -3232.97 | 27.199512 | -59.95 | 48.69 | 1.790106 |
| 4 | 10 | -3244.23 | 23.800049 | -11.26 | 14.26 | 0.599158 |
| 5 | 10 | -3269.75 | 76.573846 | -25.52 | 11.7 | 0.152794 |
| 6 | 10 | -3283.57 | 57.877381 | -13.82 | 23.26 | 0.401884 |
| 7 | 10 | -3274.13 | 58.501948 | 9.44 | 24.06 | 0.411268 |
| 8 | 10 | -3240.63 | 56.374995 | 33.5 | 20.89 | 0.370554 |
| 9 | 10 | -3228.02 | 47.980014 | 12.61 | 0.03 | 0.000625 |
| 10 | 10 | -3215.38 | 43.869397 | 12.64 | — | — |

**Figure S2.** Summary of results of STRUCTURE analysis on the Upper Yarra River sub-catchment excluding samples collected upstream of the barrier in McMahons Creek for *K* = 2 (a) and *K* = 3 (b): plots indicate proportional assignment of individuals (bars) to the colour-coded genetic clusters. The population of origin is indicated on the *x*-axis in upstream to downstream order for each stream.


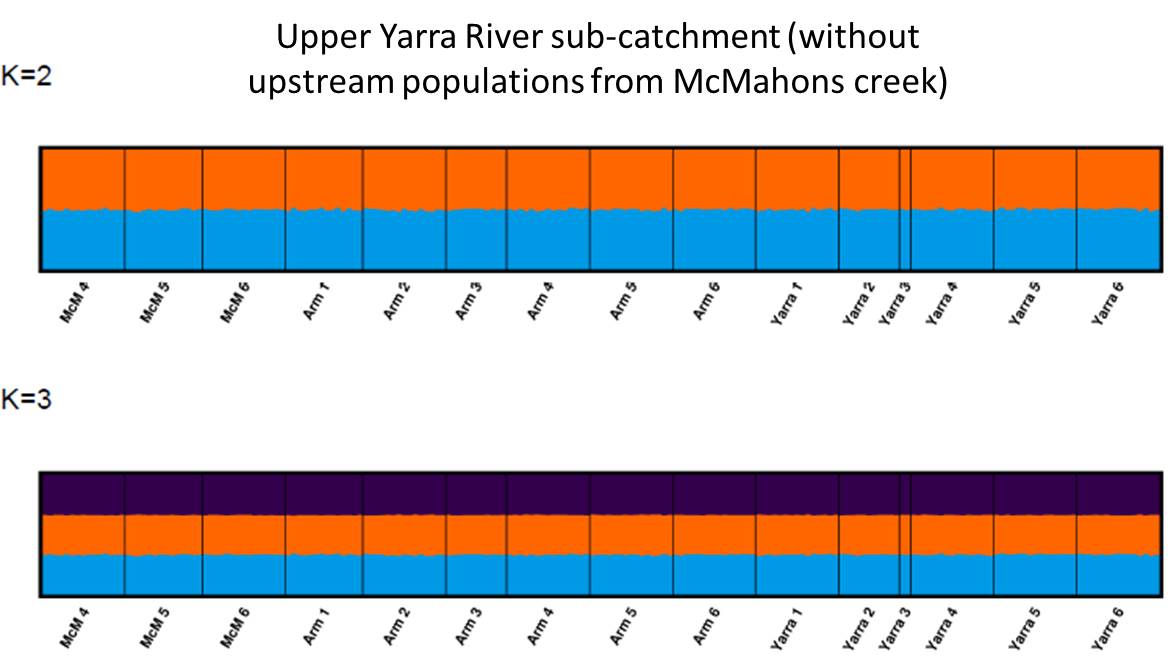


**C.1) Watts River sub-catchment**

**Table S7.** STRUCTURE analysis summary for *K* 1–10 performed on the Watts River sub-catchment dataset. The highest Delta *K* value and the highest mean LnP(K) are in bold font.

| *K* | Reps | Mean LnP(*K*) | St dev LnP(*K*) | Ln'(K) | \|Ln''(K)\| | Delta *K* |
| --- | --- | --- | --- | --- | --- | --- |
| 1 | 10 | -2286.85 | 0.254951 | — | — | — |
| **2** | **10** | **-2271.48** | **6.770492** | **15.37** | **57.78** | **8.534092** |
| 3 | 10 | -2313.89 | 76.688569 | -42.41 | 34.48 | 0.449611 |
| **4** | **10** | **-2321.82** | **8.693139** | **-7.93** | **163.73** | **18.834393** |
| 5 | 10 | -2493.48 | 20.937356 | -171.66 | 65.02 | 3.105454 |
| 6 | 10 | -2600.12 | 27.722065 | -106.64 | 52.63 | 1.898488 |
| 7 | 10 | -2654.13 | 52.352354 | -54.01 | 31.89 | 0.609142 |
| 8 | 10 | -2740.03 | 35.019488 | -85.9 | 8.88 | 0.253573 |
| 9 | 10 | -2817.05 | 54.696156 | -77.02 | 32.53 | 0.59474 |
| 10 | 10 | -2861.54 | 66.710655 | -44.49 | — | — |

**Figure S3.** Summary of results of STRUCTURE analysis on the Watts River for *K* = 2 (a) and *K* = 3 (b): plots indicate proportional assignment of individuals (bars) to the colour-coded genetic clusters. The population of origin is indicated on the *x*-axis in upstream to downstream order for each stream.

**
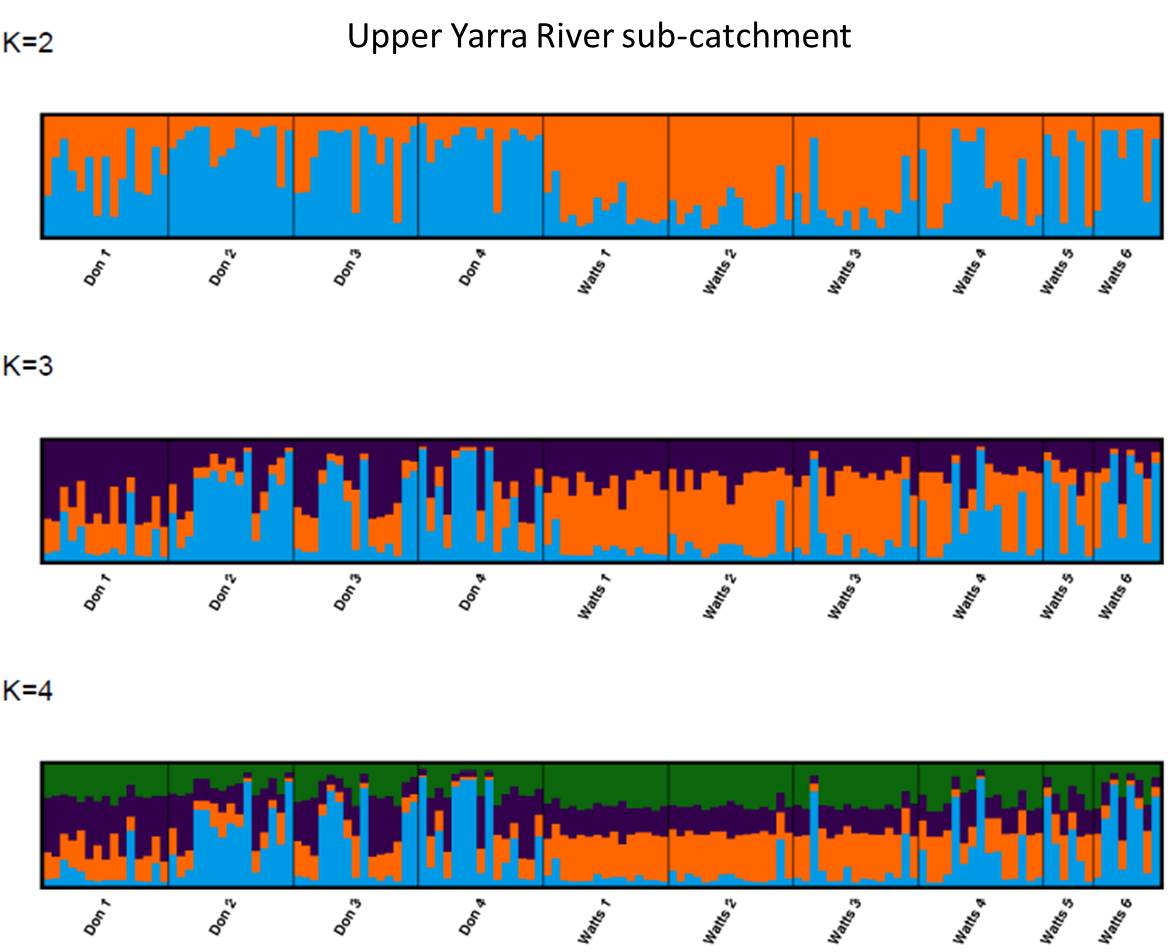
**

**Appendix S3. Genetic structure within pooled sites from the same stream and same side of each barrier**

To determine whether is was appropriate to pool samples above and below each barrier for our population genetic analyses, we investigated genetic differentiation among sampling sites from the same stream and same side of each barrier (separated by up to 5.6 km). This was achieved based on *F*_ST_ values among sampling sites with more than 10 individuals (23 out of the 28 sampling sites) using GENEPOP 4.1 (Rousset, 2008). Some caution is necessary when interpreting these results, since it has been shown that when two small samples are drawn from a population with nearly the same allele frequency distribution, they will have a positive *F*_ST_ due to sampling variation (Landguth *et al*., 2014). *F*_ST_ values were low for all comparisons (≤ 0.07), and eight of the sixteen pairs of sampling sites from the same stream and same side of each barrier were significantly differentiated (Table S8). The distribution of significant *F*_ST_ values between sampling sites was not spatially structured, suggesting they were likely transient or stochastic results due small sample size. In addition, the hierarchical STRUCTURE analysis (see above) showed that sampling sites above and below each barrier always clustered together, suggesting they are approximately panmictic units (see Appendix S2). The later was confirmed by HWE tests that showed none of the ten pooled ‘populations’ significantly departed from HWE after Significance of tests was assessed following a false discovery rate correction for multiple tests (see Table 1).

**Figure S4.** Schematic representation of the simulated linear network of 160 nodes. The vertical bar indicates the location of the barrier (between nodes 125 and 126); circles indicate every 10^th^ node, arrows indicate the 9 sampled nodes (70, 80, 90, 100, 110, 120, 130, 140 and 150) and dashed ovals show how sampled nodes were pooled to reflect the sampling design of our study.

125-126

10

60

160

20

40

50

30

Below barrier

Above barrier

**Table S8.** IBDsim simulation summary to indicate the likelihood of detecting the impact of a barrier for various population densities (*D*_e_) and number of generations since barrier installation, with eight polymorphic microsatellite markers. Summary statistics are averaged over 50 simulated datasets per dispersal scenario (*Panmixia*, *IBD* or *IBD + asymmetric migration*), density (15, 50 and 150 individuals per km²) and time since barrier installation. *% significant Wilcoxon test* = percentage of simulated datasets where Wilcoxon paired tests showed significantly higher pairwise *F*_ST_ values between pooled nodes above and below the barrier, than between the two pooled nodes below the barrier. *Ratio Ar above / Ar Below barrier* = the mean ratio of allelic richness above the barrier compared to below the barrier.

| Dispersal scenario | | Panmixia | | | IBD | | | IBD + asymmetric migration | | |
| --- | --- | --- | --- | --- | --- | --- | --- | --- | --- | --- |
| Density of individuals per km (*D*_e_) | | 15 | 50 | 150 | 15 | 50 | 150 | 15 | 50 | 150 |
| *% significant Wilcoxon test* | 0 generations | 2 | 4 | 10 | 4 | 2 | 2 | 4 | 2 | 2 |
|  | 5 generations | 60 | 30 | 22 | 14 | 2 | 6 | 4 | 2 | 2 |
|  | 10 generations | 44 | 34 | 36 | 22 | 12 | 4 | 8 | 16 | 6 |
|  | 15 generations | 56 | 50 | 36 | 20 | 10 | 12 | 20 | 16 | 4 |
|  | 20 generations | 56 | 58 | 24 | 16 | 18 | 8 | 32 | 12 | 12 |
|  | 25 generations | 64 | 50 | 44 | 32 | 18 | 22 | 20 | 16 | 10 |
|  | 50 generations | 86 | 82 | 64 | 48 | 44 | 26 | 38 | 24 | 20 |
|  | 100 generations | 80 | 100 | 94 | 60 | 64 | 44 | 44 | 40 | 30 |
| *Ratio Ar above / Ar Below barrier* | 0 generations | 0.991 | 1.003 | 0.989 | 0.951 | 0.957 | 0.948 | 0.951 | 0.957 | 0.948 |
|  | 5 generations | 0.892 | 0.923 | 0.950 | 0.946 | 0.945 | 0.951 | 0.935 | 0.917 | 0.934 |
|  | 10 generations | 0.876 | 0.902 | 0.951 | 0.932 | 0.934 | 0.940 | 0.904 | 0.961 | 0.942 |
|  | 15 generations | 0.859 | 0.889 | 0.929 | 0.906 | 0.907 | 0.930 | 0.900 | 0.915 | 0.947 |
|  | 20 generations | 0.847 | 0.874 | 0.939 | 0.929 | 0.94 | 0.936 | 0.924 | 0.918 | 0.935 |
|  | 25 generations | 0.842 | 0.878 | 0.913 | 0.899 | 0.907 | 0.935 | 0.867 | 0.91 | 0.921 |
|  | 50 generations | 0.833 | 0.844 | 0.913 | 0.822 | 0.899 | 0.918 | 0.8466 | 0.864 | 0.902 |

**Table S9.** IBDsim simulation summary to indicate the likelihood of detecting the impact of a barrier for various population densities (*D*_e_) and number of generations since barrier installation for simulations with 20 microsatellite markers. Results are averaged over 50 simulated datasets per dispersal scenario (*Panmixia*, *IBD* or *IBD + asymmetric migration*), density (15, 50 and 150 individuals per km²) and time since barrier installation. *% significant Wilcoxon test* = percentage of simulated datasets where Wilcoxon paired tests showed significantly higher pairwise *F*_ST_ values between pooled nodes above and below the barrier, than between the two pooled nodes below the barrier.

| Dispersal scenario | | Panmixia | | | IBD | | | IBD + asymmetric migration | | |
| --- | --- | --- | --- | --- | --- | --- | --- | --- | --- | --- |
| Density of individuals per km (*D*_e_) | | 15 | 50 | 150 | 15 | 50 | 150 | 15 | 50 | 150 |
| *% significant Wilcoxon test* | 0 generations | 4 | 4 | 8 | 6 | 4 | 12 | 6 | 4 | 12 |
|  | 5 generations | 86 | 58 | 32 | 38 | 8 | 8 | 16 | 2 | 4 |
|  | 10 generations | 98 | 88 | 60 | 44 | 22 | 8 | 40 | 26 | 16 |
|  | 15 generations | 98 | 94 | 74 | 70 | 40 | 26 | 52 | 34 | 20 |
|  | 20 generations | 96 | 94 | 66 | 64 | 54 | 20 | 64 | 32 | 20 |
|  | 25 generations | 96 | 90 | 90 | 82 | 50 | 40 | 70 | 52 | 24 |
|  | 50 generations | 100 | 100 | 100 | 86 | 80 | 62 | 80 | 54 | 42 |
|  | 100 generations | 100 | 100 | 100 | 98 | 100 | 96 | 78 | 78 | 74 |

**Table S10. C**omparison of observed pairwise *F*_ST_ values between pooled sites above and below each barrier, compared to pairwise *F*_ST_ values between pooled sites below barriers within each sub-catchment.

| **Waterway** | **Below-barrier pooled sites** | **Wilcoxon *P*-value** |
| --- | --- | --- |
| McMahons | McMahons and Armstrong | 0.055 |
|  | McMahons and Yarra | **0.012** |
| Armstrong | Armstrong and McMahons | 0.344 |
|  | Armstrong and Yarra | 0.320 |
| Yarra | Yarra and McMahons | 0.578 |
|  | Yarra and Armstrong | 0.473 |
| Donnellys | Donnellys and Watts | 0.371 |
| Watts | Watts and Donnellys | 0.679 |


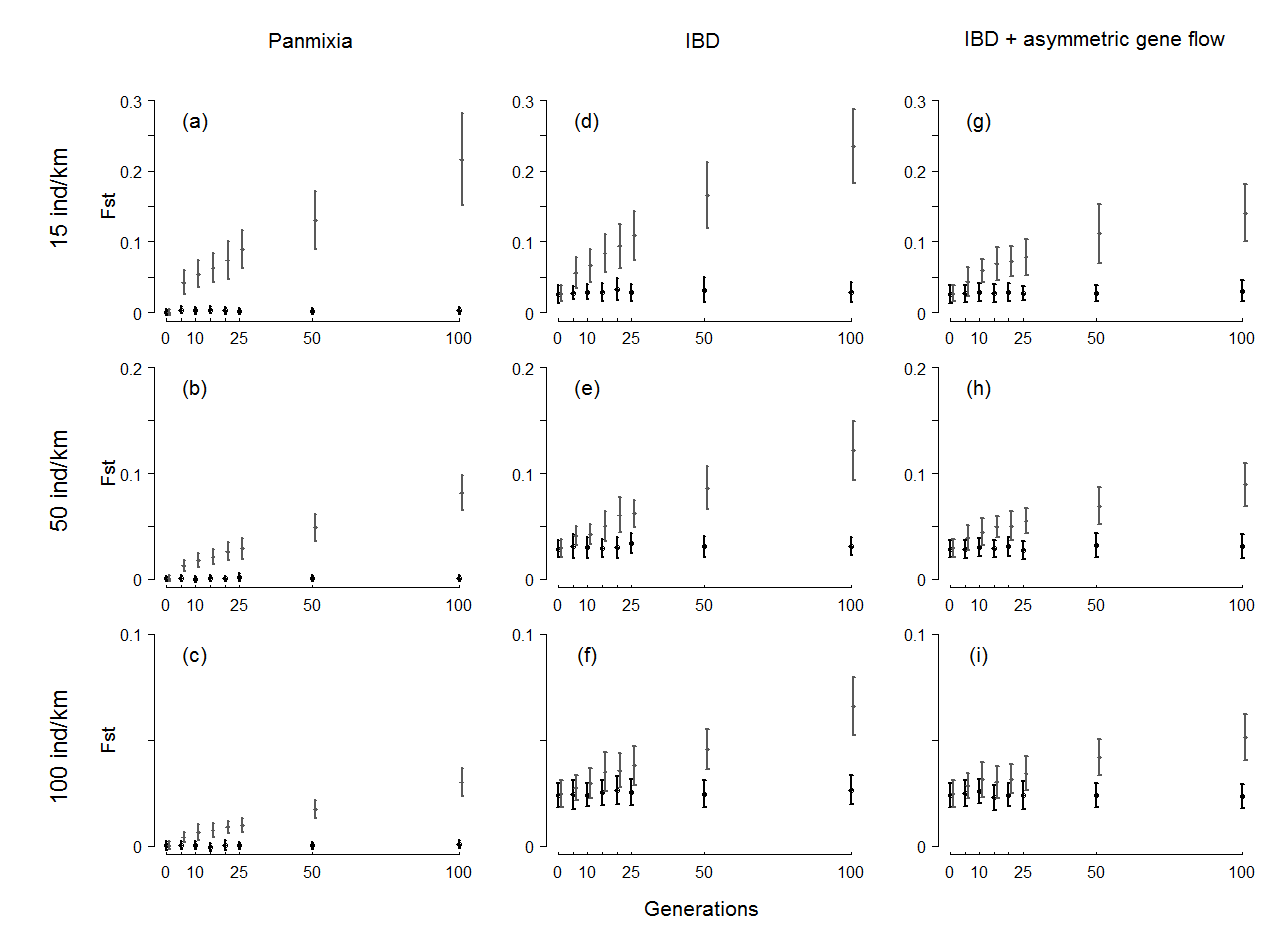
**Figure S5.** Simulated increases in population differentiation (pairwise *F*_ST_ +/- SD based on 50 simulated datasets) with time (number of generations) using 20 microsatellites markers. Pairwise *F*_ST_ values are shown (i) between pooled nodes above the barrier (nodes 130, 140 and 150) and pooled nodes below the barrier (nodes 100, 110, 120) (grey), and (ii) among two pooled nodes below the barrier (nodes 100, 110, 120 compared to nodes 100, 110, 120) (black) per dispersal scenario: *Panmixia* (a, b, c), *IBD* (d, e, f) and *IBD + asymmetric migration* (g, h, i) and effective density: 15 individuals per km (a, d, g), 50 individuals per km (b, e, h) and 15 individuals per km (c, f, i).
